# *TRPM2, PDLIM5, BCL3*, *CD14*, *GBA* Genes as Feasible Markers for Premature Coronary Heart Disease Risk

**DOI:** 10.3389/fgene.2021.598296

**Published:** 2021-05-20

**Authors:** Kriengchai Prasongsukarn, Wilanee Dechkhajorn, Surachet Benjathummarak, Yaowapa Maneerat

**Affiliations:** ^1^Pramongkutklao Hospital and College of Medicine, Bangkok, Thailand; ^2^Department of Tropical Pathology, Faculty of Tropical Medicine, Mahidol University, Bangkok, Thailand; ^3^Center of Excellence for Antibody Research, Faculty of Tropical Medicine, Mahidol University, Bangkok, Thailand

**Keywords:** familial hyperlipidemia, premature coronary heart disease, biomarker, transcriptome, predictive genes

## Abstract

**Background:** Beyond non-genetic risk factors, familial hypercholesterolemia (FH) plays a major role in the development of CHD. FH is a genetic disorder characterized by heritable and severely elevated levels of low-density lipoprotein (LDL) cholesterol, which can lead to premature cardiovascular disease, particularly familial coronary heart disease (FH-CHD).

**Method:** To explore genes indicating a risk of familial (premature) coronary heart disease (FH-CHD) development in FH, 30 Thai male volunteers were enrolled: 7 healthy controls (N), 6 patients with hypercholesterolemia (H), 4 with FH, 10 with CHD, and 3 with FH-CHD. Transcriptome data were investigated using next-generation sequencing analysis in whole blood (*n* = 3). Genes that were significantly expressed in both FH and FH-CHD, but not in N, H, and CHD groups, were selected and functionally analyzed.

**Results:** The findings revealed that 55 intersecting genes were differentially expressed between FH and FH-CHD groups. Ten of the 55 genes (*MAPK14*, *TRPM2*, *STARD8*, *PDLIM5*, *BCL3*, *BLOC1S5*, *GBA*, *RBMS1*, *CD14*, and *CD36* were selected for validation. These 10 genes play potential roles in chronic inflammation and are involved in pathways related to pathogenesis of CHD. Using quantitative real-time PCR, we evaluated the mRNA expression of the selected genes in all 30 volunteers. *TRPM2*, *PDLIM5*, *BCL3* were significantly upregulated and *GBA* was significantly downregulated in both FH and FH-CHD compared with the N, H, and CHD groups.

**Conclusion:** our preliminary investigation reveals that the *TRPM2*, *PDLIM5*, *BCL3*, and *GBA* genes may have potential for further development as predictive markers for FH-CHD.

## Introduction

Atherogenesis and the complication of coronary heart disease (CHD) involve a long preclinical process and are poorly understood. Diverse risk factors have been for CHD, including behavioral, dietary, and lifestyle factors such as smoking, fatty dietary intake, physical activity level, infection (exogenous exposure), change of endogenous blood compositions such as lipid and lipoprotein, inflammation and coagulation factors, intermediary metabolites, and oxidant markers of stress, obesity, blood pressure, and diabetes mellitus (Anand et al., 2008; Weber and Noels, 2011). Beyond these non-genetic risk factors, familial hypercholesterolemia (FH) has a major role in development of CHD (Scheuner, 2003). FH is a genetic disorder characterized by heritable and severely increased levels of low-density lipoprotein (LDL) cholesterol, which can lead to premature cardiovascular disease, particularly familial coronary heart disease (FH-CHD) (Anand et al., 2008; Weber and Noels, 2011). Several previous investigations have reported that in FH, expression of at least four genes in sterol and lipoprotein pathways, including LDL receptors, apolipoprotein (apo) B, proprotein convertase subtilisin/kexin9, and the autosomal recessive hypercholesterolemia adaptor protein, are disordered in cholesterol metabolisms, e.g., in sterol and lipoprotein pathways (Defesche, 2001; Marais, 2004). Moreover, other roles of these receptors and related receptors and other genes associated with pathophysiology of disease are of interest in evaluating the risk of developing FH-CHD. In this study, we attempted to identify alternative biomarkers for risk of FH-CHD. We conducted a cross-sectional study using Thai male volunteers, including healthy controls (N) and patients with hypercholesterolemia (H), FH, CHD, and FH-CHD. Transcriptome data were analyzed by next-generation sequencing analysis in whole blood (*n* = 3/group) and validated by quantitative real-time PCR (qPCR). We selected genes that were significantly expressed in patients with FH and FH-CHD but not in the N, H, and CHD groups to create an intersecting gene profile and then functionally analyzed selected genes. We identified 55 intersecting genes between FH and FH-CHD groups. Similar to our previous study (Maneerat et al., 2017), the expressions of intersecting genes that shared between FH and FH-CHD groups are potential for further development as predictive markers for FH-CHD in FH patients. In this study, we selected that 10 of 55 genes, *MAPK14*, *TRPM2*, *STARD8*, *PDLIM5*, *BCL3*, *BLOC1S5*, *GBA*, *RBMS1*, *CD14*, and *CD36* showed significant co-expression and potentially play roles in chronic inflammation and pathways related to pathogenesis of CHD. The selected genes were further validated in 30 volunteer samples using qPCR. The result revealed that *TRMS2, PDLIM5, BCL3, CD14*, and *GBA* genes were the most strongly associated with FH-CHD development and show potential for further application as inflammatory markers to predict the risk of FH-CHD development in Thai patients with FH.

## Materials and Methods

### Subjects

Thirty volunteers, males born to Thai parents, were enrolled in this study. Healthy volunteers who had no infections and underlying diseases or cardiovascular disease (CVD) risk factors were recruited as controls (N group; *n* = 7). Twenty-three patients were diagnosed, classified, treated and selected under supervision of a specialist (KP) at Pramongkutklao Hospital. They were classified into four groups based on their clinical manifestations according to the American College of Cardiology/American Heart Association criteria (2013) (Stone et al., 2014), and included 6 patients with high cholesterol levels [total cholesterol (TC), LDL, and high-density lipoprotein (HDL)], but with no evidence of vital organ dysfunction (H group) (Stone et al., 2014); 10 patients diagnosed with CHD (Stone et al., 2014) who were about to undergo coronary bypass grafting (CHD group); 3 patients with FH-CHD and 4 FH patients who were related to the 3 FH-CHD.

**FIGURE 1:**
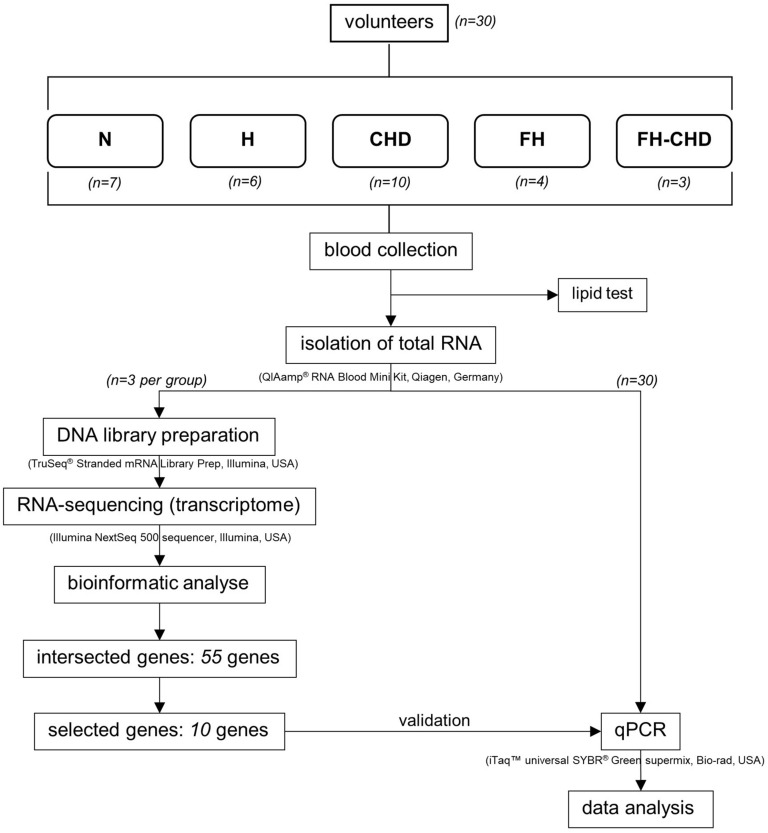
Experimental design and study population.

The workflow followed in the present study is illustrated in Figure 1. The study was performed at the Faculty of Tropical Medicine, Mahidol University. Approval for the study was considered from the Ethics Committees of the Faculty of Tropical Medicine, Mahidol University (MUTM2017-025-02), and Pramongkutklao Hospital (Q031b/59). Before enrollment, all participants were informed of the study aims, and filled an informed consent form.

### Blood Sample Collection and Methods

Heparinized blood samples (5 mL) were obtained once from healthy controls and all patients before hyperlipidemia treatment or coronary bypass grafting. Sera from 1 mL of clotted blood were collected for lipid measurement.

Packed blood cells were resuspended in 5 mL of Dulbecco’s-PBS (Wisent Inc., Quebec, Canada). Approximately 1 mL of blood suspension was immediately used to extract total RNA using QIAamp RNA Blood Mini kit (Qiagen Inc., Germantown, MD, United States). RNA samples were kept at −70°C to investigate expression of genes profiled by next-generation sequencing (NGS) and further validated by qPCR.

Lipid profiles, TC, triglycerides (TG), LDL cholesterol (LDL-c), and HDL cholesterol (HDL-c), were analyzed enzymatically using kits (Randox Laboratories Ltd., Crumlin, United Kingdom) and a biochemistry analyzer (Architect CI 16200, Abbott Laboratories, Abbott Park, IL, United States).

### cDNA Library Construction and Sequencing by NGS Technique

All total RNA samples were examined for amount and quality before analysis. Integrity of total RNA was assessed using Agilent 2100 Bioanalyzer (Agilent Technologies, Santa Clara, CA, United States). Approximately 500 ng of the total RNA from each sample was used to create individually indexed strand-specific RNA-seq libraries using TruSeq stranded mRNA library preparation kit (Illumina Inc., San Diego, CA, United States). Briefly, poly-A-containing mRNA molecules was captured using magnetic oligo (dT) beads, purified, and directed to cDNA synthesis. AMPure XP beads (Beckman Coulter Genomic, Atlanta, GA, United States) were used to separate the cDNA from reaction mix. Indexing adapters were ligated to the cDNA, and all cDNA libraries were checked for quality using an Agilent 2100 Bioanalyzer (Agilent Technologies) and quantified with DeNovix fluorometer (DeNovix Inc., Wilmington, DE, United States). The indexed sequencing libraries were pooled in equimolar quantities and subjected to cluster generation and paired-end 2 × 75 nucleotide read sequencing on an Illumina NextSeq 500 sequencer. The sequencing process was carried out at Omics Sciences and Bioinformatics Center (Bangkok, Thailand).

### Differential Expression Analyses of RNA-Seq Data and Statistical Methods

Bioinformatics analyses comprised an initial quality check of the raw data files using FASTQC software (Bioinformatics Group, Babraham Institute, Cambridge, United Kingdom). Adapter and low-quality reads were removed using Trimmomatic^1^ (Bolger et al., 2014). The filtered reads were aligned to a human reference genome using HISAT2 aligner software (Center for Computational Biology, Johns Hopkins University, Baltimore, MD, United States). StringTie (Center for Computational Biology, Johns Hopkins University) was used to assemble transcripts from RNA-seq reads that were aligned to the genome, reconstructing all isoforms expressed from each gene as well as estimates of the relative abundance of those isoforms. Subsequently, differential isoform expression among five groups was performed using edgeR program (Robinson et al., 2010) via Cuffdiff 2.0 (Trapnell et al., 2013) (University of Maryland Center for Bioinformatics and Computational Biology)^2^. Fold change ≥ 1, *p*-value ≤ 0.05, and false discovery rate (FDR) ≤ 0.05 was interpreted statistically significant. In addition, the gffcompare utility (Pertea and Pertea, 2020) (Center for Computational Biology, Johns Hopkins University, Baltimore, MD, United States)^3^. StringTie (Center for Computational Biology, Johns Hopkins University) was used to discover a novel transcript. Gene Ontology (GO) and pathway enrichment analyses was conducted using a web-based bioinformatics tool DAVID (omicX, Seine Innopolis, Le-Petit-Quevilly, France).

### Validation of Possible Marker Genes for Risk of FH-CHD by RT-qPCR

**FIGURE 2:**
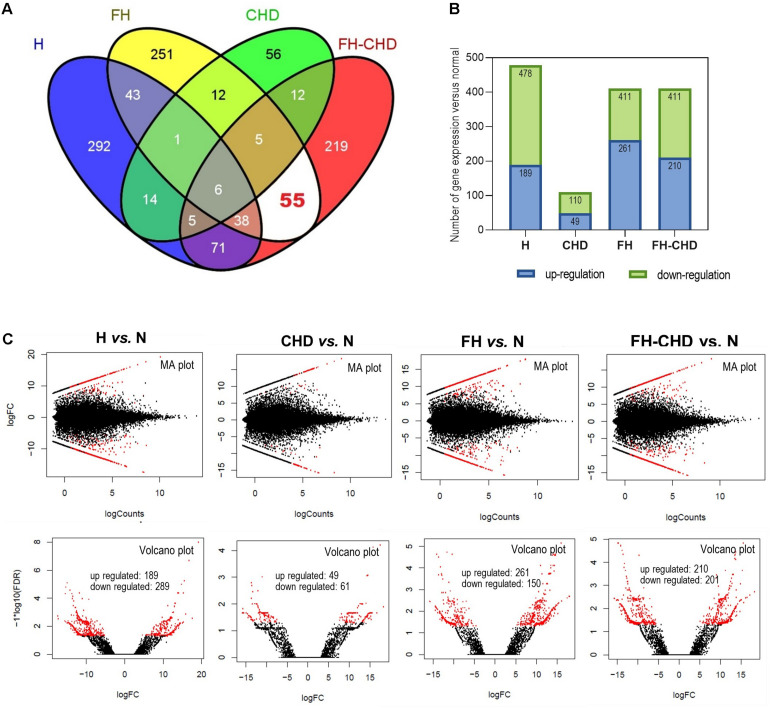
Transcriptome analysis by next-generation sequencing in four patient groups, hyperlipidemia (H), familial hyperlipidemia (FH), coronary heart disease (CHD), and familial CHD (FH-CHD) compared with healthy volunteers (N). Total RNA was extracted from blood (*n* = 3). Differentially expressed genes were performed using edgeR program via Cuffdiff 2.0 (fold change ≥ 1), *p*-value ≤ 0.05 and false discovery rate (FDR) ≤ 0.05. **(A)** Venn diagram illustrates all 1,080 differentially expressed genes, and **(B)** number of up- and downregulated genes expressed in the four patient groups compared with N. [**(C)**, upper panels] MA plots for differential expression analysis in H, CHD, FH, or FH-CHD versus N samples. The *y*- and *x*-axes represent the log fold change (FC) of gene expression and log number of genes, respectively. The red and black points in the plot indicate significant and not significant differentially expressed genes, respectively. [**(C)**, lower panels] Volcano plots of differentially expressed transcripts in H, CHD, FH, or FH-CHD versus N samples. The *y*- and *x*-axes represent the FDR value and log FC of gene expression, respectively. The filter threshold is FDR < 0.0001. The red and black points in the plot represent up- and downregulated transcripts, respectively.

Fifty-five intersecting genes, significantly expressed in both FH and FH-CHD but not in N, H, and CHD groups, were selected for further validation (Figure 2). qPCR of the mRNA expression of 10 selected genes was performed in all volunteer samples. These genes were *MAPK14, TRPM2, STARD8, PDLIM5, BCL3, BLOC1S5, GBA, RBMS1, CD14*, and *CD36*, as described in Table 1 (in bold). Table 2 lists the primers designed to amplify these genes and their expected fragment lengths. qPCR was conducted in triplicate (Susomboon et al., 2006). Each 10-μL PCR reaction contained 5 μL of iTaq universal SYBR Green supermix (BioRad Laboratories Inc., Hercules, CA, United States) mixed with 100 ng of cDNA and 10 μM of each set of forward and reverse primers (Table 2). Amplification was run in a Bio-Rad CFX96 Real-time system (BioRad Laboratories Inc.). The qPCR conditions were 95°C for 3 min, followed by 35 cycles of denaturation at 95°C for 30 s, annealing at 60°C for 30 s, and melting curve analysis at 65°C for 5 min. β-Actin (*ACTB*) (primers: forward: 5′-TCACCCACACTGTGCCCATCTACGA-3′ and reverse: 5′-CAGCGGAACCGCTCATTGCCAATGG-3′) was used to normalize the relative expression of each gene (Heid et al., 1996; Kotepui et al., 2012) and the relative expression level was calculated using the 2^–ΔΔCt^ method.

**TABLE 1:** Fifty-five intersecting genes expressed in patients in the FH and FH-CHD groups but not in the N, H, and CHD groups, including 37 significantly up-regulated and 18 down-regulated genes.

Transcript ID (Ensembl_Gene_ID)	Gene ID	DE (FH vs. N)^a^			DE (FH-CHD vs. N)^b^		
		logFC	*p*-value	FDR	logFC	*p*-value	FDR
**Upregulated genes**							
ENST00000521724.5	*CTNNA1*	13.4	<0.001	< 0.001	13.7	<0.001	<0.001
ENST00000427641.2	*NCBP2*	12.7	<0.001	0.018	13.0	<0.001	<0.001
ENST00000370793.5	*USP33*	12.6	<0.001	0.026	10.1	<0.001	0.04
ENST00000467471.5	*PPM1M*	12.1	<0.001	0.025	10.9	<0.001	0.039
ENST00000424201.6	*CACNA2D2*	12.0	<0.001	0.003	8.5	<0.001	0.049
ENST00000433797.5	*KDM6A*	11.8	<0.001	0.035	10.6	<0.001	0.041
ENST00000372384.6	*TSC22D3*	11.7	<0.001	0.03	10.5	<0.001	0.034
**ENST00000468133.5**	***MAPK14***	**11.7**	<**0.001**	**0.031**	**11.5**	<**0.001**	**0.035**
ENST00000397708.1	*MCM3AP*	11.7	<0.001	0.0049	9.8	<0.001	0.041
ENST00000633130.1	*GPI*	11.6	<0.001	0.03	12.0	<0.001	0.03
**ENST00000498430.5**	***TRPM2***	**11.5**	<**0.001**	**0.003**	**10.9**	<**0.001**	**0.04**
ENST00000498133.5	*BCL2L13*	11.4	<0.001	0.035	12.3	<0.001	0.013
**ENST00000374597.3**	***STARD8***	**11.2**	<**0.001**	**0.033**	**11.3**	<**0.001**	**0.028**
ENST00000528516.5	*LTBP3*	11.2	<0.001	0.033	10.5	<0.001	0.04
ENST00000340645.9	*GOLGB1*	11.2	<0.001	0.033	13.1	<0.001	<0.001
ENST00000495421.1	*DPH2*	11.1	<0.001	0.033	11.1	<0.001	0.038
ENST00000245960.9	*CDC25B*	11.0	<0.001	0.018	11.9	<0.001	0.024
ENST00000627233.2	*ARHGAP27*	10.9	<0.001	0.035	11.1	<0.001	0.038
ENST00000531427.5	*CUL5*	10.8	<0.001	0.035	10.5	<0.001	0.04
**ENST00000401743.6**	***CD14***	**10.7**	<**0.001**	**0.003**	**12.5**	<**0.001**	**0.029**
ENST00000580168.5	*HELZ*	10.6	<0.001	0.017	9.7	<0.001	0.042
ENST00000357364.8	*IKZF1*	10.6	<0.001	0.035	11.3	<0.001	0.003
ENST00000359741.9	*SLC39A14*	10.4	<0.001	0.021	9.6	<0.001	0.045
ENST00000487620.1	*ZNF3*	10.3	<0.001	0.035	10.7	<0.001	0.04
**ENST00000538969.5**	***CD36***	**10.2**	<**0.001**	**0.039**	**10.1**	<**0.001**	**0.048**
**ENST00000380176.7**	***PDLIM5***	**10.1**	<**0.001**	**0.037**	**10.1**	<**0.001**	**0.015**
ENST00000221232.9	*CNOT3*	10.1	<0.001	0.036	10.2	<0.001	0.04
ENST00000486484.5	*MBOAT2*	9.8	<0.001	0.038	10.5	<0.001	0.04
ENST00000436439.6	*HMGCL*	9.8	<0.001	0.038	10.0	<0.001	0.04
ENST00000543133.5	*BCL2L13*	9.6	<0.001	0.045	10.4	<0.001	0.004
ENST00000402312.7	*WDR25*	9.5	<0.001	0.047	9.8	<0.001	0.005
ENST00000575898.5	*ZNF232*	8.8	<0.001	0.04	8.8	<0.001	0.025
ENST00000464356.6	*MEF2D*	8.5	<0.001	0.027	9.6	<0.001	0.003
ENST00000513565.6	*CEP120*	8.4	<0.001	0.013	7.7	<0.001	0.033
ENST00000397053.6	*UPF2*	8.3	<0.001	0.021	7.1	<0.001	0.043
**ENST00000487394.1**	***BCL3***	**7.6**	<**0.001**	**0.033**	**8.0**	<**0.001**	**0.04**
ENST00000354958.6	*PLEC*	5.6	<0.001	0.041	7.1	<0.001	0.021
**Down-regulated genes**							
ENST00000470843.5	*RPL5*	−8.0	<0.001	0.021	−9.5	<0.001	0.004
ENST00000439657.5	*LENG8*	−9.2	<0.001	0.017	−9.1	<0.001	0.018
**ENST00000543936.5**	***BLOC1S5***	−**9.8**	<**0.001**	**0.004**	−**9.8**	<**0.001**	**0.004**
ENST00000574897.5	*NPLOC4*	−9.9	<0.001	0.038	−9.8	<0.001	0.041
ENST00000463567.5	*ZNF767P*	−9.9	<0.001	0.014	−9.9	<0.001	0.015
ENST00000514886.1	*PRMT9*	−10.1	<0.001	0.036	−10.0	<0.001	0.040
ENST00000562631.5	*ADGRG1*	−10.3	<0.001	0.024	−8.4	<0.001	0.040
**ENST00000523864.5**	***STARD8***	−**10.3**	<**0.001**	**0.036**	−**10.2**	<**0.001**	**0.040**
ENST00000264244.7	*TIMMDC1*	−10.7	<0.001	<0.001	−10.7	<0.001	0.039
ENST00000551043.5	*CNOT2*	−10.8	<0.001	0.037	−10.7	<0.001	0.041
ENST00000242848.8	*ZC3H13*	−10.8	<0.001	0.02	−8.9	<0.001	0.038
ENST00000621749.4	*RPS9*	−10.9	<0.001	0.034	−10.9	<0.001	0.038
**ENST00000368373.7**	***GBA***	−**11.1**	<**0.001**	**0.035**	−**11.0**	<**0.001**	**0.040**
ENST00000332704.5	*TBL3*	−11.6	<0.001	0.03	−11.6	<0.001	0.034
ENST00000368060.7	*MED23*	−12.1	<0.001	0.027	−12.0	<0.001	0.031
**ENST00000474820.5**	***RBMS1***	−**12.1**	<**0.001**	**0.026**	−**10.2**	<**0.001**	**0.040**
ENST00000546079.5	*CLPTM1*	−12.9	<0.001	0.015	−12.9	<0.001	0.016
ENST00000376554.8	*STK24*	−13.0	<0.001	0.02	−13.0	<0.001	0.021

*Ten of the 55 intersecting genes (in bold) were selected for further validation by quantitative real-time PCR. DE, significant differential gene expression (fold change) comparing between familial hyperlipidemia patient (FH) versus normal (N) groups (a), and familial coronary heart disease (FH-CHD) versus normal (N) groups (b). Statistical analysis was considered using edgeR program via Cuffdiff 2.0 (fold change ≥ 1), *p*-value ≤ 0.05 and false discovery rate (FDR) ≤ 0.05 was significant.*

**TABLE 2:** Primers for gene amplification in quantitative real-time PCR.

Genes	Primer sequence (5′→ 3′)	Product size (bp)	*T*_a_ (°C)	References
***MAPK14***	F: GAGCGTTACCAGAACCTGTCTC	161	60.0	Khalaf et al. (2013), Maimaiti et al. (2016), Zhou et al. (2020)
	R: AGTAACCGCAGTTCTCTGTAGGT			
***TRPM2***	F: ATTGTGAAGCGGATGATGAAGGA	158	57.0	Lin et al. (2018)
	R: ATGGTGAGGTAGGAGTGGTAGAC			
***STARD8***	F: GCAGCTTTTTGAAGGAGGCTGAT	127	60.6	*
	R: TGGGGCCAACAGATCAGAGG			
***PDLIM5***	F: CTCGCTCTTTCCGAATCCTTGC	126	60.0	*
	R: AAGCTACCGAGGAAGCCAACTG			
***BCL3***	F: GAACACCGAGTGCCAAGAAACC	121	57.0	*
	R: GCTAAGGCTGTTGTTTTCCACGG			
***BLOC1S5***	F: CCAAATGTAGAGACACAATGCGG	106	57.0	*
	R: TCCTGTTCCCTCTGTTGGAGTC			
***GBA***	F: TGCTGCTCTCAACATCCTTGCC	135	57.0	Ivankovic et al. (2016)
	R: TAGGTGCGGATGGAGAAGTCAC			
***RBMS1***	F: GCATCTCCTGTATCTGCCTACC	170	60.0	*
	R: GGCTGTAGTGACATGGTGTGCT			
***CD14***	F: CTGGAACAGGTGCCTAAAGGAC	120	60.0	Hidaka et al. (2013)
	R: GTCCAGTGTCAGGTTATCCACC			
***CD36***	F: CAGGTCAACCTATTGGTCAAGCC	119	60.0	Soriano-Romani et al. (2015a, b)
	R: GCCTTCTCATCACCAATGGTCC			
***ACTB***	F: TCACCCACACTGTGCCCATCTACGA	295	60.0	Susomboon et al. (2006), Stone et al. (2014)
	R: CAGCGGAACCGCTCATTGCCAATGG			

*The primer sequences were designed according to ORIGENE (http://www.origene.com). Ta, annealing temperature.

## Results and Discussion

### Characteristics of Healthy Controls and Patients

General descriptions and clinical characteristics of the four patient groups and controls are presented as medians and ranges (Table 3).

**TABLE 3:** Characteristics of the participants.

Group	N	FH	FH-CHD	H	CHD
Variables	(*n* = 7)	(*n* = 4)	(*n* = 3)	(*n* = 6)	(*n* = 10)
Age (years)	27	47.5	45	52.5	51.5
	(19–36)	(20–56)	(45–79)	(26–65)	(41–61)
TC (mg/dL)	182	227.5	176	194.5	164.5
	(165–187)	(171–276)	(171–232)	(148–233)	(131–233)
TG (mg/dL)	130	119.5	177	218.5	172.5
	(82–168)	(102–256)	(125–271)	(134–289)	(73–368)
HDL (mg/dL)	48	51.5	46	49	45
	(41–66)	(35–78)	(42–48)	(37–78)	(40–65)
LDL (mg/dL)	105	152	100	102.5	89
	(98–111)	(89–175)	(80–149)	(43–153)	(15–126)
WBC (10^3^/uL)	7.8	7.5	7.3	6.1	5.7
	(3.8–12)	(5.4–8.8)	(5.4–8.5)	(5.2–9.4)	(4.9–8.6)
RBC (10^3^/uL)	4.87	5.24	5.99	4.8	4.775
	(4.58–7.35)	(4.46–6.31)	(4.19–6.38)	(3.6–7.85)	(4.46–12.5)
Hb (g/dL)	14.3	14.4	13.4	14.35	14.1
	(10.9–18.2)	(13.9–15)	(13.3–16.2)	(11.2–14.5)	(12.5–16.2)
HCT (%)	41.9	42.85	42	42.25	41.5
	(32–53.7)	(39.5–43.8)	(38.3–49.7)	(32.9–44.3)	(38–46.5)
Lymphocyte (%)	35.4	38.3	28.8	33.2	35.4
	(25–50.3)	(25–47)	(27–34.5)	(21.4–42.2)	(22.3–40.7)
Monocyte (%)	5.2	4.85	7.2	6.05	6.65
	(4.6–6.9)	(4.0–6.0)	(4–8.7)	(4.5–8.8)	(5.3–9.0)

*All patients and controls were male. Data are shown as median (ranges). N, normal controls; H and CHD, patients with hyperlipidemia and coronary heart disease; respectively. FH and FH-CHD, familial hyperlipidemia and familial coronary heart disease patients, respectively. TC, total cholesterol; TG, triglyceride; HDL, high-density lipoprotein; LDL, low-density lipoprotein; WBC, white blood cells; RBC, red blood cells; Hb, hemoglobin; HCT, hematocrit.*

### Preselection of Regulatory Sequences

Transcriptome sequences of healthy volunteers (N) and patients in the H, FH, CHD, and FH-CHD were analyzed. We found approximately 500 differentially expressed genes in the patient groups compared with the healthy controls, as illustrated in a Venn diagram (Figure 2A). The number of significantly up- and down-regulated genes in the four patient groups compared with the N group are displayed in bar graphs (Figure 2B) and volcano plots (Figure 2C).

### Intersecting and Selected Genes

**FIGURE 3:**
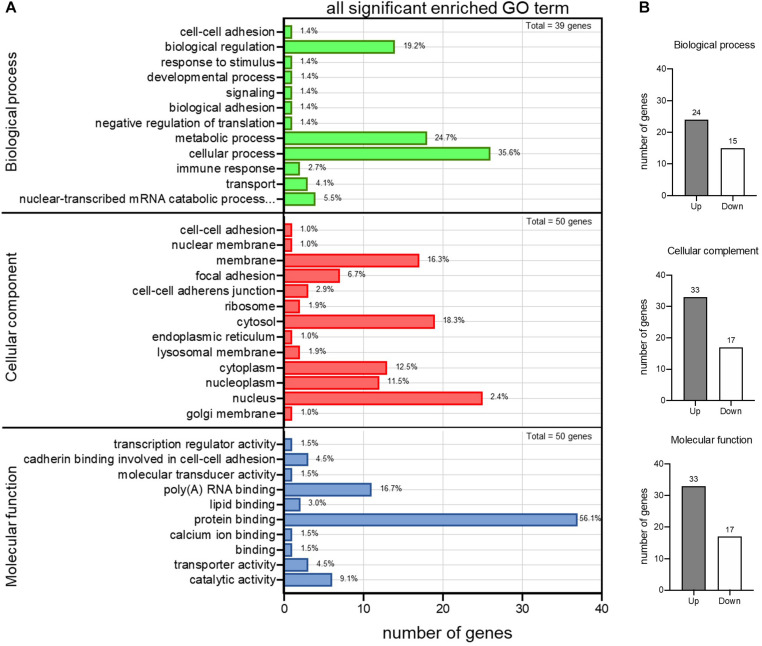
Gene Ontology (GO) enrichment analysis of differential expression of 55 intersecting genes in the familial hyperlipidemia (FH) and familial coronary heart disease (FH-CHD) versus N groups. **(A)** The ordinate represents the next level GO term of the three categories including biological process (upper panel), cellular component (middle panel), and molecular functions (lower panel). The abscissa represents the gene number under the term. **(B)** Bar graphs show number of upregulated (gray bar) and downregulated (white bar) genes in each category.

To pick up sequence group to identify predictive markers for FH-CHD risk in the FH group, we focused on the 55 genes that intersected between FH and FH-CHD (white area in Figure 2A). The intersecting gene profiles include 37 up- and 18 down-regulated genes (Table 1). Gene Ontology and pathway enrichment analyses were conducted and the results are shown in Figure 3. The GO terms were in three categories: biological process, cellular component, and molecular functions (Figure 3A). The number of up- and down-regulated genes in each category is shown in Figure 3B.

**FIGURE 4:**
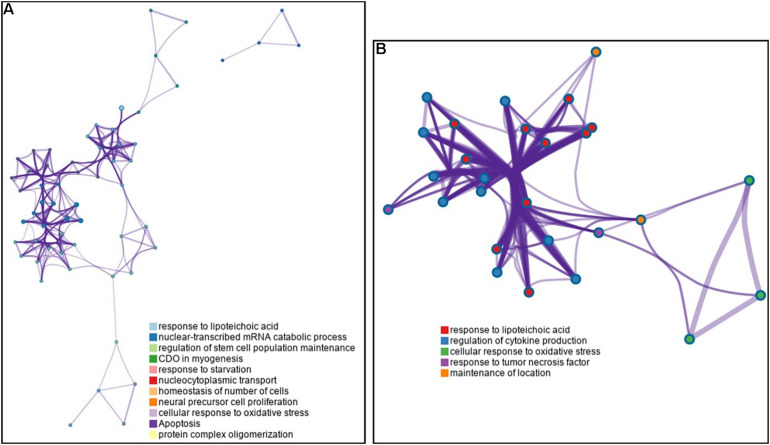
Functional enrichment analyses of differentially expressed genes at the mRNA level analyzed by Metascape pathway. **(A)** Association between the top 11 clusters and **(B)** 5 clusters of enriched terms displayed as a network enrichment analysis of 55 intersected and of 10 selected differentially expressed mRNAs, respectively. Nodes of the same color belong to the same cluster. Terms with a similarity score > 0.3 are linked by an edge. The network was visualized with Cytoscape with force-directed layout and edge bundled for clarity. The analysis was conducted using the web-based bioinformatics tool DAVID (omicX, Seine Innopolis, Le-Petit-Quevilly, France).

**FIGURE 5:**
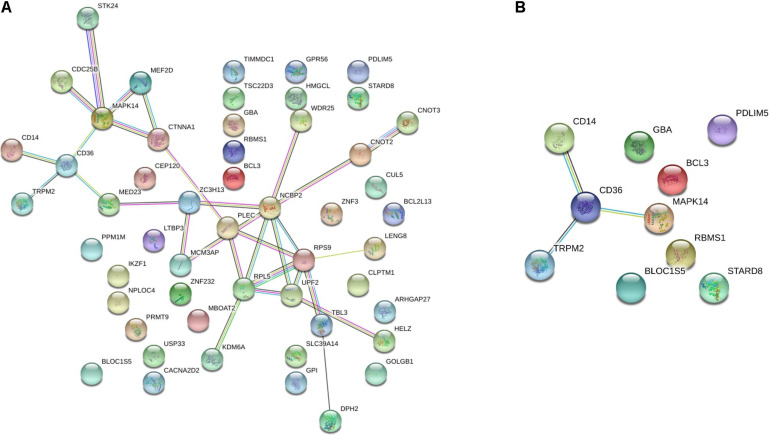
The protein–protein interaction network of **(A)** the 55 intersecting genes between familial hyperlipidemia (FH) and familial coronary heart disease (FH-CHD) groups, and **(B)** in 10 genes selected from the 55 intersecting genes using web-based bioinformatics tool: functional protein association networks analysis.

To investigate the importance of the intersecting genes, we conducted functional enrichment analyses of genes differentially expressed at the mRNA level analyzed by Metascape pathway^4^. We observed functional correlations between the top 11 clusters in the 55 intersecting genes between FH and FH-CHD; the genes were mainly involved in lipid metabolism, cellular process, oxidative stress, and inflammation (Figure 4A). Focusing on 10 differentially expressed genes, 5 clusters are displayed as the same network enrichment analysis of the 55 intersecting genes (Figure 4B). In addition, the expected protein–protein interaction networks in the 55 intersecting genes and the 10 selected genes analyzed by web-based bioinformatics tool are shown in Figures 5A,B, respectively.

### Verification of 10 Selected Genes Using RT-qPCR

**FIGURE 6:**
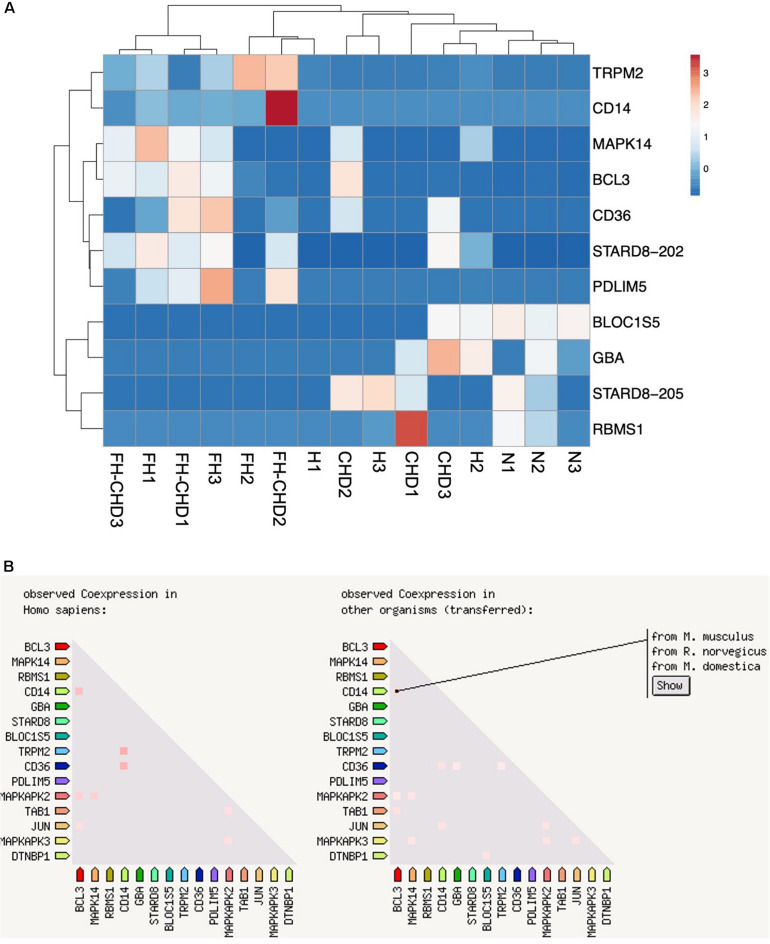
Ten selected genes from 55 intersecting genes between familial hyperlipidemia (FH) and familial coronary heart disease (FH-CHD) groups. **(A)** Heat maps of the 10 differentially expressed transcripts in the patient groups versus the normal control group (*n* = 3). **(B)** Co-expression of 10 selected genes. Note the high co-expression between *CD14* and *BCL3*, between *TRMP2* and *CD14*, and between *CD14* and *CD36* (left panel) in *Homo sapiens* and in other organisms (right panel).

**FIGURE 7:**
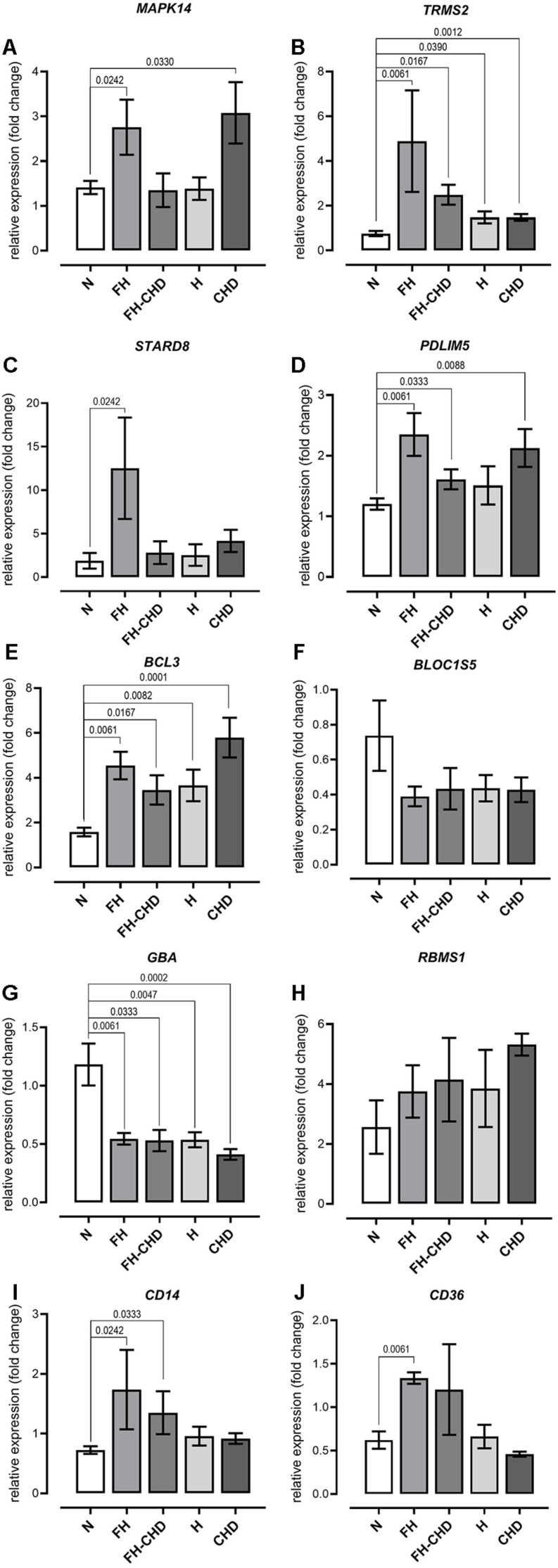
Quantitative reverse transcription PCR analysis of mRNA expression of **(A)** *MAPK14*, **(B)** *TRPM2*, **(C)** *STARD8*, **(D)** *PDLIM5*, **(E)** *BCL3*, **(F)** *BLOC1S5*, **(G)** *GBA*, **(H)** *RBMS1*, **(I)** *CD14*, and **(J)** *CD36* genes, showing altered expression in patient groups vs. controls. mRNA expression (2.0-fold change) relative to *ACTB* mRNA in RNA samples obtained from the patient groups; familial hyperlipidemia (FH; *n* = 4), familial coronary heart disease (FH-CHD; *n* = 3), hyperlipidemia (H; *n* = 6), and coronary heart disease (CHD; *n* = 10) compared with the healthy (N) group (*n* = 7). Bracketed *p*-values indicate significant differences between groups.

Based on focusing regulatory and immune response roles of target genes, we selected 10 genes potentially involved in atherogenesis, chronic inflammation, or lipid metabolism. These genes include *MAPK14, TRPM2, STARD8, PDLIM5, BCL3, BLOC1S5, GBA, RBMS1, CD14*, and *CD36*. Table 4 summarizes the selected genes and their cardiovascular syndrome (CVS)-related functions. In Figure 6, we compared the expression of these genes among groups (Figure 6A) and detected high co-expression between *CD14* and *BCL3*, between *TRMP2* and *CD14*, and between *CD14* and *CD36* (Figure 6B), which may indicate a possible further extension of our indicative synergistic markers for risk of FH-CHD. The qPCR results of relative mRNA expression (mean two-fold changes) of the 10 genes in all groups using total RNA extracted from blood of volunteers are shown in Figure 7. The mRNA expression of these genes was compared among the groups. To identify ideal biomarkers, we selected only genes that were significantly up- or down-regulated in patients with FH and FH-CHD compared with healthy individuals (not differently expressed between FH and FH-CHD). The results reveal that *TRPM2*, *PDLIM5, BCL3*, and *CD14* were up-regulated and GBA was down-regulated in both FH and FH-CHD compared with the N, H and CHD groups. These five target genes have potential as markers for risk of FH-CHD in FH.

**TABLE 4:** Description and potential functions of 10 genes selected from the 55 intersecting genes expressed in both FH and FH-CHD but not in N, H, and CHD groups.

Gene ID	Description	Function
*MAPK14*	Mitogen-activated protein kinase 14; p38 MAPK	Its activation promotes cardiomyocyte hypertrophy (Zechner et al., 1997; Liang and Molkentin, 2003), promotes myocyte apoptosis (Sharov et al., 2003) via downstream targets STAT1, CHOP, FAK, SMAD, cytochrome c, NF-κB, PTEN, and p53 (Fiordaliso et al., 2001; Ghosh et al., 2009), and regulate cardiomyocyte cytokinesis and promote cell cycle exit (Engel et al., 2006).
*TRPM2*	Transient receptor potential cation channel subfamily M member 2	The encoded protein is activated by oxidative stress and confers susceptibility to cell death (Maglott et al., 2011). Ca2^+^ entry via TRMP2 is necessary for proper cardiac function through modulation of mitochondrial oxidative signals, especially after I/R and reducing reactive oxygen species levels. TRPM2 is also protective of doxorubicin cardiomyopathy (Miller et al., 2014; Hoffman et al., 2015).
*STARD8*	StAR related lipid transfer domain containing 8; Rho-GTPase-activating-protein domain (RhoGAP)	This gene encodes a member of a subfamily of Rho GTPase activating proteins that contain a steroidogenic acute regulatory protein related lipid transfer domain. The encoded protein localizes to focal adhesions and may be involved in regulating cell morphology. START proteins are involved in several different biological processes: lipid transfer between cellular compartments; lipid metabolism, which involves START proteins that contain thioesterase catalytic activities; and signal transduction, which involves the RhoGAP START proteins (Alpy and Tomasetto, 2005).
*PDLIM5*	PDZ and LIM domain 5	*PDLIM5* encodes several splice variants, whose expression is tissue specific and temporally regulated (Zheng et al., 2010). Alternative splicing plays an important role in heart development and in the development of cardiopathies (Weeland et al., 2015). Polymorphisms in PDLIM3 (rs4861669, rs4862543) and PDLIM5 (rs1056772) were significantly associated with idiopathic dilated cardiomyopathy (IDCM) in Chinese Han patients (Wang et al., 2019)
*BCL3*	BCL3 transcription coactivator	BCL3 gene expression is induced via NF-κB and play role in cell proliferation regulation (Ohno et al., 1990; Wulczyn et al., 1992). Down regulated expression of BCL3 gene affect their transcription regulatory networks, which subsequently alter a number of biological processes in human ischemia cardiomyopathy (Herrer et al., 2015).
*BLOC1S5*	Biogenesis of lysosomal organelles complex 1 subunit 5	Component of the BLOC-1 complex, a complex that is required for normal biogenesis of lysosome-related organelles (LRO), such as platelet dense granules. Dense granules are important in platelet aggregation and play role in thrombus formation (Ambrosio et al., 2012).
*GBA*	Glucosylceramidase beta	Glucosylceramidase that catalyzes the hydrolysis of glucosylceramide/GlcCer into free ceramide and glucose within the lysosomal compartment. Thereby, GBA plays a central role in the degradation of complex lipids and the turnover of cellular membranes (Rijnboutt et al., 1991). Under specific conditions, GBA may alternatively catalyze the reverse reaction, transferring glucose from cholesteryl-beta-D-glucoside to ceramide, finally, may also hydrolyze cholesteryl-beta-D-glucoside to produce D-glucose and cholesterol (Akiyama et al., 2013; Marques et al., 2016). Alterations in the level of glucosylceramide are noted in cells and tissues in response to cardiovascular disease, diabetes, skin disorders and cancer. Reducing synthesis of glycosphingolipids with pathological effects could be a new approach for preventing cardiac hypertrophy. Overall, upregulation of glucosylceramide offers cellular protection and primes certain cells for proliferation (Messner and Cabot, 2010).
*RBMS1*	RNA binding motif single stranded interacting protein 1	This gene encodes a member of a small family of proteins, which bind single stranded DNA/RNA. These proteins are characterized by the presence of two sets of ribonucleoprotein consensus sequence (RNP-CS) that contain conserved motifs, RNP1 and RNP2, originally described in RNA binding proteins, and required for DNA binding. These proteins have been implicated in such diverse functions as DNA replication, gene transcription, cell cycle progression and apoptosis (Maglott et al., 2011). RBMS1 interact with polymerase (DNA directed), alpha 1 (Niki et al., 2000).
*CD14*	Cluster of differentiation 14 molecule	CD14 is expressed mainly by macrophages in innate immunity (Simmons et al., 1989). CD14 acts as co-receptor for Toll like receptor (TLR) 4 and MD-Z in response to lipopolysaccharide (LPS) (Kitchens et al., 2000). Increase in proinflammatory monocyte (CD14^+^CD16^+^) is an independent risk factor for CAD and plaque process (Schlitt et al., 2004). Monocyte (CD14^++^CD16^+^) and neutrophil may be involved in small to large vessel vasculitis (Corbera-Bellalta et al., 2016). Soluble CD14 from peripheral blood monocytes could be biological markers for screening and monitoring inflammatory disease activity in patients (Scherberich and Nockher, 1999).
*CD36*	Cluster of differentiation 36 molecule; fatty acid translocase	The *CD36* encoded protein is the fourth major glycoprotein of the platelet surface and serves as a receptor for thrombospondin in platelets and various cell lines. This protein may have important functions as a cell adhesion molecule. It binds to collagen (Tandon et al., 1989), thrombospondin (Silverstein et al., 1992), anionic phospholipids and oxidized LDL (Podrez et al., 2002). CD36 is a key mediator of phagocytic oxLDL (oxidized low-density lipoprotein) uptake. CD36 was significantly reduced with plaque enriched long non-coding RNA in atherosclerotic macrophage regulation (PELATON) knockdown (Hung et al., 2020). CD36 (fatty acid translocase) as a key target gene for this miRNA and showed that the induced expression of CD36 is responsible for increased fatty acid uptake, thereby causing lipotoxicity in the heart (Li et al., 2019). The heat shock protein/glucocorticoid receptor (HSP/GR) complex-mediated CD36 axis was involved in the regulation of plaque formation in atherosclerosis development in mice (Silverstein et al., 1992). High-fat food-induced metabolic disorders promote lipoproteins accumulation, oxidative stresses, and active inflammation in macrophage of the vascular wall and accordingly result in the formation of atherosclerotic plaques by affecting the expression of GC, GR, HSP, CD36, and ABCA1 (cholesterol efflux regulatory protein) (Fu et al., 2019). CD36, a scavenger receptor, was at higher levels in the serum of patients with acute coronary syndrome or chronic coronary heart disease than in normal subjects (Bai et al., 2019). CD36 mediates foam cell formation and promotes inflammatory response and oxidative stress (Park, 2014; Xu et al., 2018) and promote atherosclerosis (Bai et al., 2019).

The present study had some limitations; (1) We used whole blood to prepare transcriptome data. Therefore, our data lacks cardiac genes (reviewed in Saucerman et al., 2019) that play role in cardiovascular system but not mainly express in blood cells. In our opinion, atherosclerosis and its complication, coronary heart disease and stroke are consequence of chronic inflammation of vascular wall (Hansson, 2005). Blood contains inflammatory cells, which play an important role in atherogenesis. Therefore, blood is an accessible source particularly fitting surrogate for atherosclerotic tissue (Moore et al., 2005; Kang et al., 2006; Sharp et al., 2007). In consistence, Perisic et al., 2016 demonstrated similar gene expression profiles in carotid atherosclerosis compared between PBMC and vascular tissue sources (Perisic et al., 2016). These approaches contribute that our study using blood to investigate the transcriptome data is feasible; (2) we focused only on male volunteers to control the influence of the sex-hormone factor. Estrogens, in particular, are primary examples of female sex steroids. Previous studies in animal models, e.g., with rabbits, mice, and monkeys, have reported that estrogen has protective effects in CVD. Earlier evidence has also indicated that estrogen protects women against CHD pre-menopause (reviewed in Arnal et al., 2007). Estrogen ameliorates lipidaemia by up-regulating LDL-receptor production. In addition, estrogen attenuates inflammation in the atherosclerotic plaque. It is exerted via the sex hormone receptors on various inflammatory cells in the plaques, including reduced LDL oxidation, EC activation and the adhesion of neutrophils and monocytes to the endothelial lining, and impedes nitric oxide activity (Boese et al., 2017). We expect that our preliminary findings will help further studies to find appropriate biomarkers to predict CHD in both male and female Thai hyperlipidemia patients; (3) we illustrate our findings with low statistical power due to the small genetic sample size and single-center analysis. Further studies with a larger sample size and multi-center analysis in genotypic and phenotypic expressions of the 10 potential genes will be needed to clarify our current findings; (4) We conducted this study using a single ethnic background cohort. Ethnicity is a source of health inequalities. In particular, ethnic inheritance influences the occurrence rates of different cardiovascular disorders (Scarborough et al., 2010). In this study, we performed a small preliminary study only in Thai ethnicities to elucidate appropriate biomarkers to predict FH-CHD. We expect that further studies in larger sample sizes and multi-center Thai male and female populations will help to confirm the feasibility of using the FH-CHD biomarker in Thai ethnicities. Moreover, further meta-analysis studies using publicly available transcriptomic data from different ethnic populations to compare and share appropriate CHD biomarker is expected to be more reliable and advantageous; (5) Several current studies into the genetic basis of coronary heart disease have intensively reported the discovery and aggregation of genetic variants from multiple genes from lipid and other pathologic pathways (Yao et al., 2015; Glicksberg et al., 2019; Cohain et al., 2021). Based on our current NGS data, we are continuing to investigate the variant genes observed only in FH and FH-CHD patient groups, to identify potential predictive markers.

Taken together, based on previous knowledge and our findings, we suggest that among 10 potential markers of FH-CHD risk, *TRMS2, PDLIM5, BCL3, CD14*, and *GBA* genes were the most strongly correlated with development of FH-CHD. These show potential for further application as inflammatory markers for the risk of FH-CHD development in Thai patients with FH.

## Data Availability Statement

The datasets presented in this study can be found in online repositories. The names of the repository/repositories and accession number(s) can be found below: NCBI BioProject, accession no: PRJNA663423.

## Ethics Statement

The studies involving human participants were reviewed and approved by The Ethics Committees of the Faculty of Tropical Medicine, Mahidol University, Bangkok, Thailand and Pramongkutklao Hospital, Bangkok, Thailand. The patients/participants provided their written informed consent to participate in this study.

## Author Contributions

KP chose healthy control, patients with hyperlipidemia, familial hyperlipidemia, coronary heart disease (CHD), familial CHD, and performed their coronary bypass grafting. YM and WD were responsible for laboratory work including blood collection and RNA extraction. YM and WD analyzed the NGS data. SB designed primers and conducted the qRT-PCR assays and analyses. WD worked on data analysis and statistical calculations. YM conceived the study and prepared the manuscript. All authors discussed the results, and read and approved the final manuscript.

## Conflict of Interest

The authors declare that the research was conducted in the absence of any commercial or financial relationships that could be construed as a potential conflict of interest.

## Footnotes

1 http://www.usadellab.org/cms/?page=trimmomatic

2 https://www.genepattern.org/modules/docs/Cuffdiff/7

3 https://ccb.jhu.edu/software/stringtie/gffcompare.shtml

4 https://metascape.org/gp/index.html#/main/step1
